# Development and psychometric evaluation of a Dutch-translated shorter Breast Cancer Treatment Outcome Scale (Dutch BCTOS-13)

**DOI:** 10.1186/s41687-018-0085-y

**Published:** 2018-12-03

**Authors:** Gerson M. Struik, Frank W. de Jongh, Erwin Birnie, Jean-Philippe Pignol, Taco M. Klem

**Affiliations:** 1 Department of Surgery, Franciscus Gasthuis and Vlietland, PO Box 10900, 3004 BA Rotterdam, The Netherlands; 2 Department of Statistics and Education, Franciscus Gasthuis and Vlietland, PO Box 10900, 3004 BA Rotterdam, The Netherlands; 3 Department of Genetics, UMC Groningen, University of Groningen, PO Box 30001, 9700 RB Groningen, the Netherlands; 4 Department of Radiation Oncology, Erasmus MC - Cancer Institute, PO Box 5201, 3008 AE Rotterdam, The Netherlands; 5 Department of Radiation Oncology, Dalhousie University, 5820 University Avenue, Halifax, NS B3H1V7 Canada

**Keywords:** BCTOS, Breast cancer, Breast conserving treatment, Cosmesis, Clinical validity, Dutch

## Abstract

**Purpose:**

To create a Dutch translated short version of the Breast Cancer Treatment Outcome Scale (BCTOS) and validate it in patients who have completed both breast conserving surgery and adjuvant radiotherapy.

**Methods:**

The BCTOS consists of items comparing the treated with the untreated breast. After forward and backward translation, we tested the BCTOS-12 plus 5 additional items. Two-hundred breast cancer patients treated with breast conserving therapy (BCT) between January 2016 and December 2017, were asked to complete the BCTOS items twice with a 2 week interval. The EORTC QLQ-BR23 breast and arm symptoms subscales were completed once in parallel. Feasibility was assessed by missing or non-unique answer rates and content validity with floor and ceiling effect analysis. Construct validity was evaluated with 1) principal component analysis (PCA) 2) convergent validity and 3) known groups comparison (clinical validity differentiating between patients with and without locoregional side effects). From all potential items with good feasibility, content and construct validity, items were selected for the Dutch BCTOS based on clinical validity. The relation to the EORTC QLQ-BR23 subscales and reliability was tested for the new Dutch BCTOS.

**Results:**

Hundred and one of 200 (50.5%) approached patients participated in this study, with follow-up after surgery ranging from 5 to 29 months. Feasibility was high (1.5% missing answers). Content validity testing showed a floor effect > 20% in all 17 items. PCA showed that all items loaded well (> 0.4) into the assigned subscale and revealed two distinct subscales: cosmesis and function. Based on clinical validity, item “breast shape” was replaced by “breast elevation/position” and “overall skin appearance”. Very good clinical validity (Cohen’s d = 1.38) was found for the new Dutch BCTOS-13. Correlation to the EORTC QLQ-BR23 subscales was high (ICC = 0.65–0.85) for both subscales. Test-retest reliability (Cohen’s d = 0.105) and internal consistency (Cronbach’s α =0.90) were excellent.

**Conclusions:**

Psychometric evaluation of a newly developed Dutch BCTOS-13 questionnaire in BCT patients showed excellent results, that were slightly better than the original BCTOS-22 and the shortened BCTOS-12. The good clinical validity makes the BCTOS-13 a useful tool to identify patients with unfavourable cosmetic and functional outcomes, requiring specific attention.

**Electronic supplementary material:**

The online version of this article (10.1186/s41687-018-0085-y) contains supplementary material, which is available to authorized users.

## Introduction

Breast conserving therapy (BCT), consisting of a wide local excision and adjuvant radiotherapy, is equally effective as breast amputation in early stage breast cancer patients [1]. Furthermore, it has cosmetic and functional benefits, which are directly related to patients’ quality of life [2, 3]. Oncoplastic techniques are increasingly used to further improve the cosmetic outcome of the surgical treatment [4]. The effect of high conformal or partial irradiation techniques on cosmetic outcome of adjuvant radiotherapy has been investigated in several clinical trials [5–12] showing benefit in most of these studies. With the excellent oncologic outcomes in this patient group, patient reported outcome measures (PROMs) are increasingly important to indicate healthcare quality and compare different surgical and radiotherapeutic techniques [13–15]. Brouwers et al. found that PROMs can be used to identify breast cancer patients who experience a heavy burden of late side-effects (≥3 months after completion of the radiation treatment), requiring specific attention. The use of PROMs instead of a standard outpatient clinical visit potentially spares visits in those patients with good cosmetic and functional outcomes [16].

Among others, the validated 22-item English Breast Cancer Treatment Outcome Scale (BCTOS) [17] is a questionnaire that is widely used [14, 18–23]. Its outcome is based on the comparison of the treated and untreated breast by the patient. It is clearly structured, comprehensive and assesses the most important aspects of morbidity after BCT. The questionnaire includes a cosmetic, functional and breast sensitivity subscale. The original BCTOS-22 was validated in patients after completing all treatment, so also including radiotherapy in the majority of patients. Therefore, it is widely used as a PROM in radiotherapy clinical trials [23–25].

However, for the best adoption of a questionnaire, besides being valid and comprehensive it should also be concise. Therefore, a shortened 12-item English version (BCTOS-12) has been developed and tested recently. The study by Hennigs et al. [22] showed good validity, without loss of information. In this shortened version the 12 items are assigned to two, instead of three, subscales: aesthetic and functional status. In contrast to the original version, the shortened BCTOS-12 was only validated in patients within a week after surgery.

The aim of the present study is to create a Dutch translated short version of the Breast Cancer Treatment Outcome Scale (BCTOS) and validate it in patients who have completed both breast conserving surgery and adjuvant radiotherapy. A translated version of the English BCTOS-12 was used in this study. As the aim of this study is to create a version of the BCTOS that is specifically valid for use in patients after adjuvant radiotherapy, we included 5 additional exploratory items in the cosmetic subscale, to anticipate for any differences in outcome in our population compared to the study by Hennigs [22]. The additional items were selected on the expectation that cosmesis, and more specifically skin outcome, could be influenced by the adjuvant radiotherapy. Selection of items to be included in the final questionnaire will be based on psychometric properties, specifically focussing on the clinical validity to identify patients with locoregional side effects related to the BCT.

## Methods

The protocol of this cross-sectional validation study was reviewed by the TWOR regional medical research ethics committee (MREC), Rotterdam, the Netherlands. Ethical clearance for this study was granted (2018–16).

### Study population

Our study population consisted of breast cancer patients treated with BCT, including adjuvant radiotherapy in the vast majority. All patients had their surgical treatment in the period January 2016 to December 2017 in the Franciscus Gasthuis and Vlietland, a large secondary teaching hospital in Rotterdam, the Netherlands. We chose this population as we are aiming to create a PROM that is valid to assess both surgical ánd radiotherapy outcomes. All participants were at least 18 years of age and able to understand the Dutch language. Patients that underwent major reconstructive surgery were excluded, since specific questionnaires have been developed for this group [26]. Bilateral breast cancer and mastectomy patients were excluded, as a comparison between the treated and untreated breast is not possible in these patients. Finally, patients with (planned) locoregional breast cancer treatment during data collection were excluded. All patients gave their written informed consent for study participation.

### Study instruments: BCTOS-12, additional exploratory items, the European organization for research and treatment of cancer (EORTC) QLQ-BR23 questionnaire

We used the Hennigs’ BCTOS-12 questionnaire [22] as basis for the items to be tested in our study. The patients in their study were asked to complete the BCTOS questionnaire within a week after surgery. In our study patients will have completed both surgery as well as radiotherapy at a minimum of 2–3 months after surgery. A previous study by Heil et al. [20] found that functional outcome as scored with the BCTOS is stable over time, while cosmetic outcome is not. Therefore, we anticipated on differences in cosmetic outcomes of our study population compared to that of Hennigs’ study [22], by adding five exploratory items to the cosmetic subscale. The three cosmetic subscale items that showed high factor loadings in the original BCTOS (but were removed when the shortened BCTOS-12 was developed) were included for exploration (“breast size”, “breast elevation/position” and “fit of clothing”). Two protocol specific items, that were expected to specifically capture radiotherapy related skin toxicity and fibrosis, were also included for exploration ( “overall skin appearance” and “overall breast appearance”). These five exploratory items are also in use in ongoing breast radiotherapy clinical trials [27–29] to investigate cosmetic outcome.

The final questionnaire consists of 17 items, which are assigned to two subscales; 12 in a cosmetic subscale, 5 in a functional subscale. For details see Additional file 1. Patients were asked to rate each item of the questionnaire on a four-point scale evaluating the differences between the treated and the untreated breast (1 = no difference, 2 = slight difference, 3 = moderate difference, 4 = large difference). The score for each subscale is the unweighted mean of the ratings over all items belonging to that subscale. A higher score reflects less symmetry between the treated and the untreated breast and is therefore considered a measure of poor status.

An additional questionnaire containing 7-items of the EORTC QLQ-BR23 (Additional file 2) was completed once for external convergent validity testing. We chose this questionnaire as it is widely used and available and validated in the Dutch language [30]. We only used the two relevant subscales of the EORTC QLQ-BR23 assessing the same determinants as the BCTOS; breast symptoms subscale to compare with the BCTOS cosmetic subscale and arm symptoms subscale to compare with the BCTOS functional subscale.

### Development of the Dutch BCTOS

The Dutch BCTOS was developed according to the adaptation process as described by Bullinger et al. [31]. A forward translation of the 17 items from English into Dutch was performed by three Dutch native speakers with extensive knowledge of the English language. The aim was to obtain conceptual equivalence using simple language, rather than achieving a literal translation. Any difficulties in the translation were discussed with the principal investigator until consensus was reached on an optimal Dutch phrasing. A backward translation to English was performed by two native English speakers who are fluent in Dutch. These backward translations were compared with the original items, and any differences were analysed. Finally, necessary changes in the formulation of the Dutch version were made in order to arrive at the exact original English formulation after backtranslation.

The pilot version (Additional file 3) was tested in five patients treated with BCT in our centre. They were asked to comment on readability and comprehension of the questionnaire. No relevant comments were made, so no additional changes were made hereafter.

### Study design

Included patients were invited to complete the 17 items BCTOS pilot questionnaire twice with a two week interval for psychometric data collection. EORTC QLQ-BR23 breast and arm symptoms subscales were added in parallel with the first BCTOS for external convergent validation purposes.

### Psychometric evaluation

Psychometric evaluation consisted of the following analyses. These analyses were based on pairwise complete data of items in the first BCTOS and the EORTC QLQ BR-23. Data from the second BCTOS was only used for test-retest analysis.

#### Feasibility

Missing (no option chosen) or non-unique responses (> 1 option chosen) were considered invalid and reported as n and percentages. Feasibility of the questionnaire was evaluated by response rates and missing answer percentages. Questionnaires with more than one invalid response were excluded from further analyses. There is no recommendation on handling missing data by the authors of the original BCTOS or BCTOS-12. With only 4 out of 105 patients excluded in our study, it is unlikely that this has impacted the outcome of this study.

#### Content validity

Floor and ceiling effects were measured by calculating the percentage of patients scoring the minimum (floor) and maximum (ceiling) score for each item [26].

#### Construct validity

##### 1. PCA

Since the original BCTOS-22 consists of 3 subscales (cosmetic status, functional status and breast specific pain) and Hennigs’ shortened BCTOS-12 uses 2 subscales (aesthetic and functional status) we considered both a 2 and 3 factor solution. Two criteria were used to assess the validity of both options: Kaiser criterion (Eigen values> 1) and a scree plot analysis.

To identify items that did not load distinctly on a single factor, a principal component analysis with orthogonal Varimax rotation was performed on the original factor loadings. We used the same criteria as the original BCTOS development study [17] to select items eligible for exclusion from the questionnaire: items with a low (< 0.4) factor loading on their main factor and a high loading on the other factor (> 0.3).

##### 2. Convergent validity

Convergent validity was assessed on the item and subscale level. Convergent validity of an item was confirmed when item-total correlation with the assigned subscale was high (ICC > 0.4) and discriminant validity was confirmed when an item had an ICC with the assigned subscale that was > 2 standard errors higher than its ICC with the other subscale .

##### 3a. Known group comparison

It was hypothesized that the new Dutch BCTOS would be clinically valid by identifying patients with radiotherapy or surgery related toxicity. Toxicity scoring as performed by the treating physicians (i.e. surgeons/radiation oncologists) during clinical follow-up visits and recorded in the electronic patient file, according to RTOG/EORTC [32], LENTSOMA [33], and CTCAE [34] toxicity scales, was used in our analysis. The clinical validity per item was assessed with the effect size [35] and an unpaired Student’s t test for both all grades and ≥ grade 2 toxicity.

##### 3b. *Selection of the items to form the new Dutch BCTOS*

As the aim of this study is to create a Dutch version of the BCTOS that is clinically valid for use in patients treated with both breast conserving surgery and adjuvant radiotherapy, final decision on the selection of items was made based on the known group comparison analysis. The shortened BCTOS-12 set of items was the starting point. If any of the 5 additional exploratory items showed good content and convergent validity ánd showed better clinical validity than any of the retained original items, this could result in a replacement of that item. This decision was made after a meeting of the research group, before continuing further analysis for the new set of BCTOS items.

##### 4. Relationship to the EORTC QLQ-BR23

Convergent validity of the conceptual related subscales of the new BCTOS (cosmesis and function) and the acknowledged EORTC QLQ-BR23 (breast symptoms and arm symptoms) was assessed using interclass correlation coefficient (ICC model [36]: two-way mixed-a fixed number of instruments and all instruments are used in all patients (thereby, instruments and patients are two sources of data variation); single measures-as based on the individual patient data in the study instead of group averages; consistency- scores are measured on different scales).

#### Reliability

Two aspects of reliability were evaluated. To assess whether items evaluate the same concept (cosmesis, function), internal consistency of subscale items was measured using Cronbach’s α, which should exceed 0.70 [37].

Test–retest reliability was assessed with the intraclass correlations (ICC model [36], two-way mixed - a fixed number of instruments and all instruments are used in all patients (thereby, instruments and patients are two sources of data variation); single measures - as based on the individual patient data in the study instead of group averages; absolute agreement - scores are measured on the same scale) and effect size (Cohen’s d calculated as d = mean difference (retest-test)/SD_test_ [38] and interpreted as 0.01 ≤ d < 0.2 = very small, 0.2 ≤ d < 0.5 = small, 0.5 ≤ d < 0.8 = medium, 0.8 ≤ d < 1.2 = large, 1.2 ≤ d < 2.0 = very large, and d ≥ 2.0 = huge) [39].

Descriptive statistics were used to describe the study sample. All statistical tests were performed with IBM SPSS Statistics version 24.0, with two-sided *p*-values below 0.05 considered statistically significant. In case of one missing answer, that item was not included in the subscale average score.

## Results

### Study sample

One hundred one of the 200 (50.5%) approached patients participated in this study by completing at least the first BCTOS questionnaire and the EORTC QLQ-BR23 questionnaire. Patient characteristics are shown in Table 1.

**Table 1:** Patient characteristics

Total patients	101
Median age, years (range)	61 (39–86)
Type of surgery	
Lumpectomy only	5 (5.0%)
Lumpectomy + SNB	81 (80.2%)
Lumpectomy + ALND	6 (5.9%)
Lumpectomy + SNB + volume replacement (level 2)	5 (5.0%)
Lumpectomy + ALND + volume replacement (level 2)	2 (2.0%)
Wedge resection for Paget’s disease	2 (2.0%)
Follow-up since surgery, months, median (range)	14.9 (5–29)
History of breast radiotherapy	97 (96.0%)
Axillary radiotherapy	6 (5.9%)
Locoregional side-effects^a^	
Any surgery or radiotherapy related	56 (61%)
Radiotherapy related	29 (32%)
Surgery related	13 (14%)
Radiotherapy and surgery related	14 (15%)
Grade 1	26 (28%)
Grade 2	24 (26%)
Grade 3	6 (7%)

*SNB* Sentinel node biopsy, *ALND* Axillary lymphnode dissection

^a^side effects not reported in 9 patients

#### Feasibility

Missing answer rate for the BCTOS was 1.5%, ranging from 0% (item 2, 5-7, 13–17) to 4.0% (item 3, 8, 10). Four patients were excluded for further analysis because of > 1 missing answers in any of the questionnaires.

#### Content validity

The proportion minimum score of “1” (floor effect) was 0.46 (SD = 0.18), ranging from 0.24 (item 11) to 0.83 (item 17). A floor effect > 20% occurred in 17/17 items.

The proportion of maximum score of “4” (ceiling effect) was 0.08 (SD = 0.04), ranging from 0.01 (item 17) to 0.14 (item 3). A ceiling effect > 20% occurred in 0/17 items.

This means there is a floor effect and no ceiling effect in all the BCTOS items tested.

#### Construct validity

##### 1.PCA

Based on the Kaiser criterion and scree plot analysis, a two or three factor solution would be possible: Eigenvalue of 2.5 for 2 subscales with a cumulative explained variance of 58.6%, or Eigenvalue of 1.1 for 3 subscales with a cumulative explained variance of 65.3%. The difference between the two and three factor solution was that the items “breast texture”, “nipple appearance”, “scar tissue”, “breast sensitivity” and “breast tenderness” were forming a separate subscale in the three factor solution. However, with the Eigen value being only slightly > 1 and the difficulty to create three clinically relevant subscales based on the pattern of factor loadings, we opted for the two factor solution for further analysis.

The principal component analysis with two factor solution (Table 2) including all 17 items shows that all of the tested items loaded well (> 0.4) into the subscale we assigned them to. The item “breast swelling” (Dutch: Zwelling van de borst) loaded well (> 0.4) in both subscales. None of the items was eligible for exclusion.

**Table 2:** Principal component analysis. Items and factor loadings of all items explored for the Dutch BCTOS

Item	Subscale	
	Cosmesis	Function
1 Breast size^a^	0.77	0.01
2 Breast texture (hardening)	0.67	0.37
3 Nipple appearance	0.63	0.17
4 Breast shape	0.69	0.19
5 Breast elevation/position^a^	0.65	0.32
6 Scar tissue	0.63	0.04
7 Breast swelling	0.50	0.45
8 Fit of bra	0.80	0.20
9 Breast sensitivity	0.73	0.18
10 Fit of clothing^a^	0.69	0.03
11 Overall breast appearance^a^	0.85	0.10
12 Overall skin appearance^a^	0.72	0.27
13 Breast tenderness	0.48	0.40
14 Arm heaviness	0.19	0.90
15 Shoulder discomfort	0.11	0.81
16 Arm discomfort	0.19	0.89
17 Arm swelling	0.06	0.85

Displayed are factor loadings after varimax rotation. Underlining of the factor loading indicates to which subscale it was assigned. ^a^additional items that were not included in the study by Hennigs [22]

All five additional cosmetic items had high factor loadings (range 0.65–0.85) for cosmesis and low factor loadings for function (range 0.01–0.32). Cronbach’s α if item deleted, was very similar for all cosmetic items (range 0.91–0.92). Also, all the additional exploratory items were highly correlated (ICC > 0.6) with at least one of the shortened BCTOS-12 cosmetic items.

##### 2. Convergent validity

Convergent validity was confirmed for all 17 items. Discriminant ability was also confirmed for all items.

##### 3a.Known group comparison

Of the retained original items, “breast shape” showed poorest clinical validity to differentiate between patients with and without locoregional radiotherapy or surgery related side effects, with very small effect sizes (Cohen’s d = 0.34 for any grade and 0.11 for ≥ grade 2).

Of the five additional items tested, “overall skin appearance” showed a large effect size for side effects of any grade (Cohen’s d = 1.18) and medium effect size for ≥ grade 2 (Cohen’s d = 0.71). “Breast elevation/position” showed large effect size for side effects of any grade (Cohen’s d = 1.00) and medium effect size for ≥ grade 2 (Cohen’s d = 0.62). The other items showed only small effect sizes, meaning little clinical validity.

##### 3b. Selection of the items to form the new Dutch BCTOS-13

With the content and convergent validity being acceptable to good for all items, our item selection was fully based on clinical validity of the single items. Therefore, we decided to remove the original item “breast shape” and to add the exploratory items “breast elevation/position” and “overall skin appearance” to form the new Dutch BCTOS-13. The new Dutch BCTOS-13 showed very good clinical validity (mean score of 2.08 (SD = 0.60) in patients with vs. 1.42 (SD = 0.39), Cohen’s d = 1.38 in patients without any locoregional side effects, and 2.22 (SD = 0.58) vs. 1.60 (SD = 0.51), Cohen’s d = 1.17 for ≥grade 2 side effects. The BCTOS-13 was used for further analysis (Tables 3, 4 and 5). In comparison, the EORTC QLQ-BR23 questionnaire showed a smaller effect size in this regard (Cohen’s d = 1.03 and 0.32).

**Table 3:** Principal component analysis. Items and factor loadings of the all items in the new Dutch BCTOS-13

Item	Subscale	
	Cosmesis	Function
1 Breast texture (hardening)	0.78	0.27
2 Nipple appearance	0.63	0.10
3 Breast elevation/position	0.63	0.31
4 Scar tissue	0.70	−0.05
5 Breast swelling	0.58	0.41
6 Fit of bra	0.78	0.18
7 Breast sensitivity	0.77	0.11
8 Overall skin appearance	0.79	0.20
9 Breast tenderness	0.61	0.30
10 Arm heaviness	0.22	0.90
11 Shoulder discomfort	0.14	0.81
12 Arm discomfort	0.24	0.88
13 Arm swelling	0.12	0.85

Displayed are factor loadings after varimax rotation. Underlining of the factor loading indicates to which subscale it was assigned

**Table 4:** Correlations (ICCs[95% CI]) of the new Dutch BCTOS-13 with arm and breast symptoms EORTC QLQ-BR23 subscales

EORTC QLQ-BR23 subscale	BCTOS-13 cosmesis	BCTOS-13 function	BCTOS-13 total
Breast symptoms	0.65 [0.52-0.75]	0.59 [0.44-0.70]	0.74 [0.63-0.82]
Arm symptoms	0.37 [0.18-0.53]	0.85 [0.78-0.90]	0.62 [0.47-0.73]
Total	0.58 [0.43-0.70]	0.75 [0.65-0.83]	0.75 [0.64-0.82]

all correlations are significant at the 0.001 level

**Table 5:** Reliability analysis showing the test-retest effect sizes and intra-class correlation coefficient (ICC) and internal consistency

	Test re-test reliability (*n* = 93)					Correlation,	Internal consistency ^a^	
BCTOS item	Test		Re-test		Effect size	ICC [95% CI]		Cronbach’s α
	Mean	SD	Mean	SD	Cohen’s *d*	Test re-test, *n* = 93	Item-subscale total^ab^*n* = 101	*n* = 101
Cosmetic subscale^a^	1.95	0.69	1.88	0.61	0.112	0.89 [0.84–0.93]		0.89
1 Breast texture (hardening)	2.06	0.92	2.03	0.83	0.034	0.82 [0.74–0.87]	0.79 [0.69–0.85]	
2 Nipple appearance	1.93	1.08	1.86	1.05	0.074	0.85 [0.78–0.90]	0.60 [0.45–0.72]	
3 Breast elevation/position	1.86	1.00	1.81	0.92	0.053	0.76 [0.66–0.84]	0.65 [0.52–0.75]	
4 Scar tissue	2.12	0.94	2.09	0.86	0.034	0.82 [0.74–0.88]	0.60 [0.45–0.71]	
5 Breast swelling	1.62	0.91	1.50	0.79	0.132	0.83 [0.75–0.89]	0.62 [0.39–0.76]	
6 Fit of bra	1.84	0.93	1.71	0.90	0.130	0.76 [0.66–0.84]	0.75 [0.65–0.83]	
7 Breast sensitivity	2.15	0.99	2.05	0.94	0.099	0.72 [0.60–0.80]	0.70 [0.56–0.79]	
8 Overall skin appearance	1.85	0.88	1.70	0.78	0.172	0.79 [0.69–0.86]	0.77 [0.67–0.84]	
9 Breast tenderness	2.11	0.97	2.07	0.93	0.043	0.79 [0.71–0.86]	0.63 [0.49–0.74]	
Functional subscale^a^	1.46	0.73	1.43	0.67	0.032	0.90 [0.86–0.93]		0.90
10 Arm heaviness	1.49	0.87	1.51	0.85	0.024	0.84 [0.78–0.89]	0.92 [0.88–0.95]	
11 Shoulder discomfort	1.49	0.93	1.48	0.87	0.011	0.83 [0.75–0.88]	0.84 [0.77–0.89]	
12 Arm discomfort	1.60	0.89	1.55	0.87	0.058	0.86 [0.80–0.90]	0.91 [0.86–0.94]	
13 Arm swelling	1.24	0.58	1.19	0.47	0.090	0.87 [0.81–0.91]	0.78 [0.69–0.85]	
All items^a^	1.80	0.62	1.74	0.56	0.105	0.91 [0.87–0.94]		0.90

^a^analysis performed for the new BCTOS-13 ^b^analysis performed on test

##### *4.* Relationship to EORTC QLQ-BR23

Convergent validity testing showed that correlation to the EORTC QLQ BR23 subscales was high for both the functional (ICC = 0.85 (95%CI [0.78–0.90]) and the new cosmetic subscale (ICC = 0.65 (95%CI [0.52–0.75]) (Table 4).

#### Reliability (Table 5)

Mean BCTOS-13 scores were 1.81 (SD = 0.62) in the test versus 1.74 (SD = 0.56) in the re-test and test-retest effect size was very small, Cohen’s d = 0.105. There was a high correlation between the test and re-test BCTOS-13 scores, ICC was 0.91 (95%CI[0.87–0.94]). A high correlation was also found on a subscale and single-item level (Table 5).

Internal consistency was high; Cronbach’s α was 0.90 for all Dutch BCTOS-13 items, 0.89 for the cosmetic subscale and 0.90 for the functional subscale.

## Discussion

The aim of this study was to validate a Dutch translation of the BCTOS with a specific focus on the clinical validity in patients treated with breast conserving surgery ánd adjuvant radiotherapy.

The original BCTOS was developed to create a measure of perceived aesthetic and functional status after breast-conserving surgical treatment (BCT) and radiotherapy. It was validated in patients after completion of all locoregional treatment. The BCTOS is clearly structured, with the patient comparing the treated with the untreated breast. Although the BCTOS-22 is widely used, a shorter version, with any redundant items removed might be more practical and further improve clinical adoption. We used the recently validated shortened version, the BCTOS-12, as a base for our translated version. As our goal was to create a PROM valid to differentiate between favourable and unfavourable BCT outcomes, we tested five additional items in the cosmetic subscale. By doing this, we anticipated for specifically better capturing unfavourable radiotherapy outcomes in our study population. The reason to do this was that we included patient after completing all locoregional treatment with a broad range of 5 to 29 months follow-up after surgery, instead of 1 week post-surgery in the study by Hennigs [22]. The additional exploratory items were selected on the expectation that cosmesis, and more specifically skin outcome, could be influenced by the adjuvant radiotherapy. We did not expect any differences in functional outcomes, as very few patients received axillary radiotherapy or axillary lymph node dissection. Also, a previous study by Heil et al. [20] found that functional outcome as scored with the BCTOS is stable over time, while cosmetic outcome is not.

Psychometric evaluation of the proposed new Dutch BCTOS items showed comparable to slightly better results than both the original version and the shortened BCTOS-12. Feasibility was high, with an overall missing answer rate of only 1.5% (compared to 5.5% for the English BCTOS-12) [22] and construct and convergent validity was good. Clinical validity testing resulted in the removal of one item from the BCTOS-12 (“breast shape”). Two of the additional exploratory items tested (“breast elevation/position” and “overall skin appearance”) showed specific value in differentiation between favourable and unfavourable BCT outcome and were added to form the new Dutch BCTOS-13. Consistent with the study by Hennigs [22], this questionnaire comprises two subscales: cosmesis and function.

Reliability was high with only a very small test-retest effect size (Cohen’s d = 0.105). Internal consistency was high with a Cronbach’s α of 0.89 for the cosmetic subscale and 0.90 for the functional subscale. This is comparable to the original BCTOS-22 questionnaire that showed an Cronbach’s α of 0.89 for cosmesis and a 0.91 for function. Notably, internal consistency of our Dutch BCTOS-13 is higher than the English BCTOS-12, which showed an α of 0.86 and 0.81 respectively.

Correlation to the EORTC BR23 subscales was stronger for our BCTOS-13 (strong for functional subscale to arm symptoms and moderate for cosmetic subscale to breast symptoms) compared to both the English original BCTOS-22 and the shortened BCTOS-12 (weak to moderate for both subscales). The higher internal consistency and correlation to the EORTC BR23 that was found in our study than in the BCTOS-13 study, might be explained by the timing of filling out the questionnaire. We hypothesize that patients are more consistent after getting used to certain symptoms or treatment outcomes (reduction of post-surgery complaints, perhaps adaptation and/or coping). This higher consistency will also increase correlation between the two conceptual comparable questionnaires (i.e. BCTOS and EORTC BR23).

Content validity analysis showed that in this study there was a floor effect> 20% in all items. This effect was most prominent in the functional subscale, with a mean proportion of minimum scores of 71%, compared to 34% in the cosmetic subscale. In the studies by Stanton et al. and Hennig et al. no floor/ceiling effect analysis was reported. However, the distribution of scores was comparable in the original BCTOS and the shortened BCTOS-12, which would probably result in a similar floor effect in those studies, although not reported.

The floor effect that was found, could be considered as a limitation of the BCTOS. However, with the good cosmetic and functional outcomes in BCT patients this finding was expected to occur in our study, consistent with other studies testing the BCTOS in BCT patients [17, 22]. In our study only 8 patients underwent axillary lymph node dissection and in 6 patients the axilla was irradiated. Results might be different in a high risk patient population undergoing breast conserving surgery. We would not recommend changing a scale that is already widely used, as this will impede comparison between studies. A better option would be to use the categories to interpret scores as suggested by Hennigs [22]: good (1.00–1.75); intermediate (1.76–2.50), fair (2.51–3.25), and poor (3.26–4.00) outcome. More important here is the good clinical validity of the Dutch BCTOS-13 that was demonstrated, which supports clinical use of the BCTOS to differentiate between favourable and unfavourable BCT outcomes.

Another limitation of our study was that we only used the two relevant EORTC subscales (breast symptoms and arm symptoms), instead of the complete EORTC QLQ-BR23 questionnaire. We chose to specifically focus on locoregional outcome, thereby limiting patient burden for participation. Doing this is common, related studies also analyzed correlation on a subscale level. However, therefore we were not able to draw any conclusions on the correlation between cosmetic and functional outcomes with overall quality of life. The previously found strong correlation between functional outcome and overall quality of life should be confirmed in subsequent research. Furthermore, our study population was quite homogenous regarding the received treatment. The vast majority underwent lumpectomy with sentinel node biopsy and adjuvant whole breast irradiation. Results might be different in other patient groups undergoing axillary lymph node dissection and/or irradiation, level 2 oncoplastic surgery or partial breast irradiation more frequently. Further validation should be performed in these specific patient groups. On the other hand, our study population was very heterogeneous regarding time after surgery, ranging from 5 to 29 months. This means that all of our patients completed locoregional treatment. Therefore, in contrast to the study by Hennigs [22], our questionnaire has now been validated for use in both breast cancer surgery and adjuvant radiotherapy.

Implications of our study findings are the recommendation to use the Dutch BCTOS-13 questionnaire as a PROM in all breast cancer research assessing cosmetic and functional outcome after adjuvant radiotherapy in the Netherlands. Clinical validity is superior to the commonly used EORTC QLQ-BR23 for this specific patient group. The BCTOS-13 could be used to identify patients with unfavourable BCT cosmetic and functional outcomes that require specific attention. Furthermore, in patients with favourable outcome, using the BCTOS-13 potentially reduces the need for clinical visits to assess BCT outcome.

In conclusion, we developed a shorter Dutch version of the BCTOS (Dutch BCTOS-13). Despite the reduced number of items, psychometric evaluation showed excellent results that were slightly better than the original BCTOS-22 and the shortened BCTOS-12. The design makes it suitable for assessment of cosmetic and functional outcomes in patients treated with breast conserving surgery ánd adjuvant radiotherapy.

## Additional files

Additional file 1: Breast Cancer Treatment Outcome Scale self-administered questionnaire. (DOCX 15 kb)

Additional file 2: EORTC QLQ - BR23 Dutch questionnaire (arm symptoms and breast symptoms). (DOCX 17 kb)

Additional file 3: Dutch Breast Cancer Treatment Outcome Scale (BCTOS). (DOCX 15 kb)
