# Supplementary material for: Development and psychometric evaluation of a Dutch-translated shorter Breast Cancer Treatment Outcome Scale (Dutch BCTOS-13)
Source: J Patient Rep Outcomes. 2018 Dec 3;2:60. doi: 10.1186/s41687-018-0085-y (PMC6291412; doi:10.1186/s41687-018-0085-y)
Supplement: Supplementary file 1 — Breast Cancer Treatment Outcome Scale self-administered questionnaire. (DOCX 15 kb) [file 41687_2018_85_MOESM1_ESM.docx]

**Additional file 1**

**Breast Cancer Treatment Outcome Scale self-administered questionnaire**

To be filled by the patient with the following instruction: “You have been treated with breast conserving therapy for breast cancer. As you know, a reason for choosing this treatment is to keep the treated breast looking and feeling as close to normal as possible. Your opinion concerning the appearance of your breast that received the radiation treatment and the arm function on that side is valuable to us. This form will be confidential. Circle the number next to the word that best describes how your breast looks now. Circle only one answer per question.”

**How would you rate the Difference between treated and untreated breast?**

| **items** | **None** | **Slight** | **Moderate** | **Large** |
| --- | --- | --- | --- | --- |
| 1   Breast size | 1 | 2 | 3 | 4 |
| 2   Breast texture (hardening) | 1 | 2 | 3 | 4 |
| 3   Nipple appearance | 1 | 2 | 3 | 4 |
| 4   Breast shape | 1 | 2 | 3 | 4 |
| 5   Breast elevation (how high the breast is) | 1 | 2 | 3 | 4 |
| 6   Scar tissue | 1 | 2 | 3 | 4 |
| 7   Breast swelling | 1 | 2 | 3 | 4 |
| 8   Fit of bra | 1 | 2 | 3 | 4 |
| 9   Breast sensitivity | 1 | 2 | 3 | 4 |
| 10 Fit of clothing |  |  |  |  |
| 11 Overall breast appearance | 1 | 2 | 3 | 4 |
| 12 Overall skin appearance | 1 | 2 | 3 | 4 |
| 13 Breast tenderness | 1 | 2 | 3 | 4 |
| 14 Arm heaviness | 1 | 2 | 3 | 4 |
| 15 Shoulder discomfort | 1 | 2 | 3 | 4 |
| 16 Arm discomfort | 1 | 2 | 3 | 4 |
| 17 Arm swelling | 1 | 2 | 3 | 4 |
