# Supplementary material for: Development and psychometric evaluation of a Dutch-translated shorter Breast Cancer Treatment Outcome Scale (Dutch BCTOS-13)
Source: J Patient Rep Outcomes. 2018 Dec 3;2:60. doi: 10.1186/s41687-018-0085-y (PMC6291412; doi:10.1186/s41687-018-0085-y)
Supplement: Supplementary file 2 — EORTC QLQ - BR23 Dutch questionnaire (arm symptoms and breast symptoms). (DOCX 17 kb) [file 41687_2018_85_MOESM2_ESM.docx]

**Additional file 2**

**EORTC QLQ - BR23 Dutch questionnaire (arm symptoms and breast symptoms)**

**Studienummer:**

**Datum van invullen:**

Soms zeggen patiënten dat ze de volgende klachten of problemen hebben. Wilt u aangeven in welke mate u deze klachten of problemen gedurende de afgelopen week heeft ervaren aan de geopereerde (en bestraalde) zijde?

| **Gedurende de afgelopen week:** | **Helemaal niet** | **Een beetje** | **Nogal** | **Heel erg** |
| --- | --- | --- | --- | --- |
| 1.Had u pijn in uw arm of schouder? | **1** | **2** | **3** | **4** |
| 2.Heeft u een gezwollen arm of hand gehad? | **1** | **2** | **3** | **4** |
| 3.Was het moeilijk om uw arm naar omhoog of opzij te bewegen? | **1** | **2** | **3** | **4** |
| 4.Heeft u pijn gehad in het gebied van uw aangedane borst? | **1** | **2** | **3** | **4** |
| 5.Was het gebied van uw aangedane borst gezwollen? | **1** | **2** | **3** | **4** |
| 6.Was het gebied van uw aangedane borst overgevoelig? | **1** | **2** | **3** | **4** |
| 7.Heeft u huidproblemen gehad in het gebied van uw aangedane borst (bijv. jeukerig, droog of schilferachtig)? | **1** | **2** | **3** | **4** |
