# Supplementary material for: Development and psychometric evaluation of a Dutch-translated shorter Breast Cancer Treatment Outcome Scale (Dutch BCTOS-13)
Source: J Patient Rep Outcomes. 2018 Dec 3;2:60. doi: 10.1186/s41687-018-0085-y (PMC6291412; doi:10.1186/s41687-018-0085-y)
Supplement: Supplementary file 3 — Dutch Breast Cancer Treatment Outcome Scale (BCTOS). (DOCX 15 kb) [file 41687_2018_85_MOESM3_ESM.docx]

**Additional file 3**

**Dutch Breast Cancer Treatment Outcome Scale^*^ (BCTOS)**

^*^ In Nederlands: Borstkanker behandeling uitkomst schaal

**Studienummer:**

**Datum van invullen:**

In te vullen door de patiënte met de volgende instructie: "U onderging een borstsparende behandeling voor borstkanker. Zoals u weet is een reden voor de keuze van deze behandeling, om de behandelde borst er zo normaal mogelijk uit te laten zien en te laten voelen. Uw mening over het uiterlijk van uw borst die de geopereerd (en bestraald) is en de functie van de arm aan die zijde, is waardevol voor ons. Dit formulier is vertrouwelijk. Omcirkel het nummer bij het woord dat het beste beschrijft hoe uw borst er nu uit ziet. Omcirkel slechts één antwoord per vraag.

Hoe zou u het *verschil* tussen de behandelde en onbehandelde borst beschrijven?

|  | **Geen** | **Licht** | **Matig** | **Groot** |
| --- | --- | --- | --- | --- |
| 1   Borst grootte | 1 | 2 | 3 | 4 |
| 2   Borst textuur (verharding) | 1 | 2 | 3 | 4 |
| 3   Hoe uw tepel eruit ziet | 1 | 2 | 3 | 4 |
| 4   Vorm van de borst | 1 | 2 | 3 | 4 |
| 5   Borst hoogte/positie (of de borst ‘hangt’ of juist opgetrokken is) | 1 | 2 | 3 | 4 |
| 6   Littekenweefsel | 1 | 2 | 3 | 4 |
| 7   Zwelling van de borst | 1 | 2 | 3 | 4 |
| 8   Pasvorm van de BH | 1 | 2 | 3 | 4 |
| 9   Mate van gevoel in de borst | 1 | 2 | 3 | 4 |
| 10 Pasvorm van kleding | 1 | 2 | 3 | 4 |
| 11 Hoe de borst er als geheel uitziet | 1 | 2 | 3 | 4 |
| 12 Hoe de huid er als geheel uitziet | 1 | 2 | 3 | 4 |
| 13 Overgevoeligheid van de borst | 1 | 2 | 3 | 4 |
| 14 Zwaar gevoel van de arm | 1 | 2 | 3 | 4 |
| 15 Schouderklachten | 1 | 2 | 3 | 4 |
| 16 Armklachten | 1 | 2 | 3 | 4 |
| 17 Zwelling van de arm | 1 | 2 | 3 | 4 |
